# Biomechanically Valid Clear Aligner Therapy for Premolar Extraction Case: A Case Report and Literature Review

**DOI:** 10.1155/crid/1856931

**Published:** 2025-10-22

**Authors:** Yimeng Xu, Bingguo Kou, Xiayu Zhang, Yirong Hu, Yue Hui, Siyu Lan, Zexu Gu

**Affiliations:** State Key Laboratory of Oral & Maxillofacial Reconstruction and Regeneration, National Clinical Research Center for Oral Diseases, Shaanxi Clinical Research Center for Oral Diseases, Department of Orthodontics, School of Stomatology, The Fourth Military Medical University, Xi'an, Shaanxi, China

**Keywords:** clear aligners, evidence-based orthodontics, extraction treatment, tooth movement protocol

## Abstract

This case report details the successful orthodontic management of an adult female with skeletal Class II malocclusion, protrusive facial profile with perioral muscle tension, and mild crowding using clear aligners following extraction of four first premolars. To address the inherent challenges of “roller-coaster effects,” a biomechanically appropriate protocol was implemented including rational case analysis and selection, as well as attachment design, anchorage control, overcorrection strategy, and tooth movement pattern. The treatment process successfully avoided refinements and the “roller-coaster effects,” accomplishing the entire therapeutic plan with the initial series of clear aligners. This study demonstrates the individualized biomechanical protocols to bridge discrepancies between virtual treatment predictions and real-world therapeutic efficacy, particularly in premolar extraction cases where uncontrolled tipping threatens predictability.

## 1. Introduction

Clear aligner (CA) therapy has become increasingly favored by patients of all ages for esthetic appeal, comfort, and longer intervals between check-ups. With the advancement of CA technology, CA has evolved from initially addressing mild malocclusions and simple dental alignment to currently managing various complex malocclusions while achieving multiple types of tooth movement. However, due to characteristics such as low elastic modulus of aligner materials, material fatigue, and aligner aging, elastic recovery of aligners can only provide minimal orthodontic forces and the sustained orthodontic forces for tooth movement decay rapidly, leading to deficiencies such as inadequate root movement, weak anchorage control, and suboptimal tooth movement efficiency during treatment [1, 2]. Since clinical application of CA, suboptimal treatment efficiency has remained a critical factor limiting therapeutic outcomes. Moreover, there exists significant variability in treatment effectiveness and efficiency across different case types and tooth movement patterns [3].

In CA therapy involving teeth extractions, two primary challenges arise. Firstly, CA therapy demonstrates limited efficiency in achieving bodily movement and torque control of teeth during extraction cases. Secondly, the aligners create low-stress zones around extraction sites, resulting in inadequate control over adjacent teeth. These biomechanical limitations frequently lead to “off-track” phenomenon and “roller-coaster effects”—characterized by lingual inclination and extrusion of incisors, distal tipping of canines, and mesial inclination with intrusion of posterior teeth during anterior teeth retraction [4–13]. Consequently, premolar extraction cases requiring anterior retraction remain one of the persistent technical challenges in CA therapy [14]. A clinical survey revealed that approximately two-thirds of orthodontists intentionally avoided using CAs for premolar extraction cases to enhance tooth movement predictability and optimize treatment outcomes, primarily due to concerns about the “roller-coaster effects” and “off-track” followed by multiple refinement phases [15].

To address “roller-coaster effects” and recurrent refinements after aligner “off-track” resulting from inefficient tooth movement in CA treatment, researchers have dedicated substantial efforts to enhance clinical outcomes, aiming to reduce chairside time, minimize clinical visits, and achieve predictable treatment outcomes. However, existing studies on extraction-based CA therapy predominantly offer fragmented solutions targeting isolated biomechanical factors, with limited evidence regarding the systematic integration of multiple strategies to maximize movement accuracy and eliminate refinements in clinical practice. This critical knowledge gap persists despite growing technical innovations. This study bridges the gap through a premolar extraction case report, synthesizing evidence from existing literature on improving tooth movement accuracy in extraction cases and validating these strategies in clinical practice. By demonstrating the successful application of integrated design principles, we explored design strategies to prevent refinements in extraction case with CA, providing clinicians with biomechanically valid workflows for enhanced clinical outcomes.

## 2. Case Presentation

### 2.1. Diagnosis and Etiology

A 24-year-old female patient complained of protruding anterior teeth affecting facial profile esthetics after eruption of permanent teeth. The patient had no negative oral habits and denied a history of dental treatment, systemic diseases, or family genetic diseases at her first consultation.

**Figure 1:**
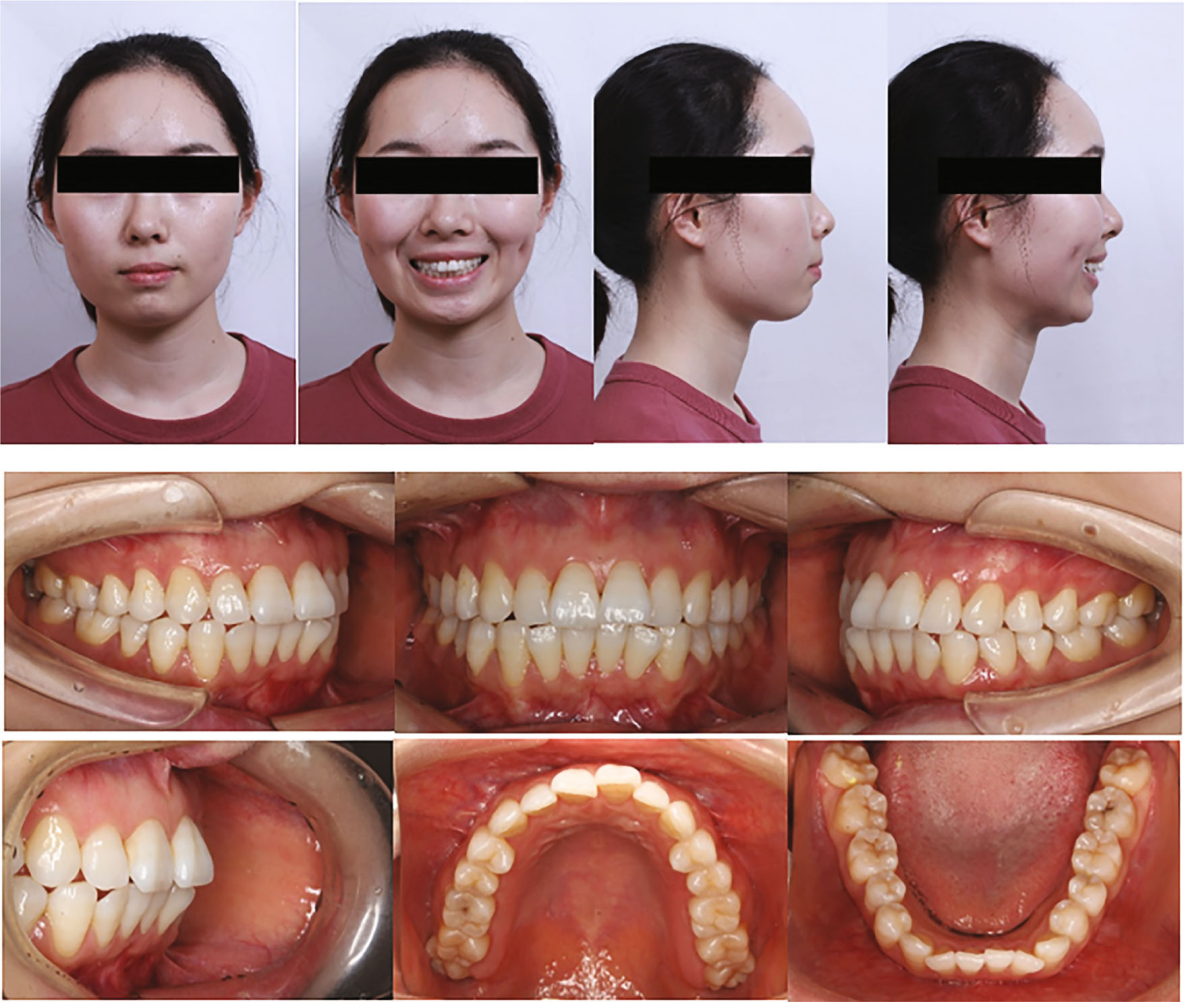
Pretreatment facial and intraoral photographs.

**Figure 2:**
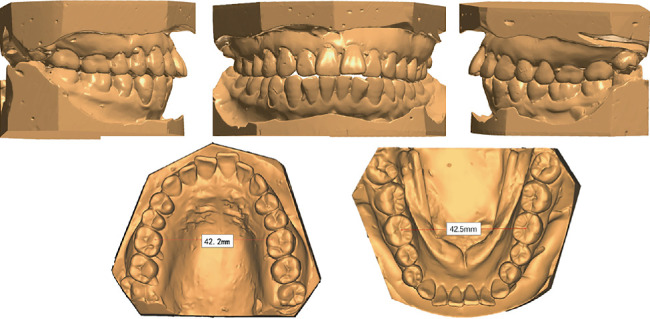
Pretreatment dental casts.

The patient presented a convex profile with mild asymmetry. Slight tension in perioral muscles was observed during lip closure. Mild crowding was present in both upper and lower dentitions. Bilateral canine and molar relationship were both Class I relationship. The overjet of the anterior teeth was approximately 2 mm, and the overbite of the anterior teeth was approximately 1 mm. The transverse widths of the upper and lower dental arches were essentially consistent. The lower midline was deviated to the right side by approximately 1 mm. Periodontal examination revealed mild gingival recession, a “black triangle” between upper central incisors, visible root morphology in lower anterior teeth, and a thin gingival biotype (Figures 1 and 2).

**Figure 3:**
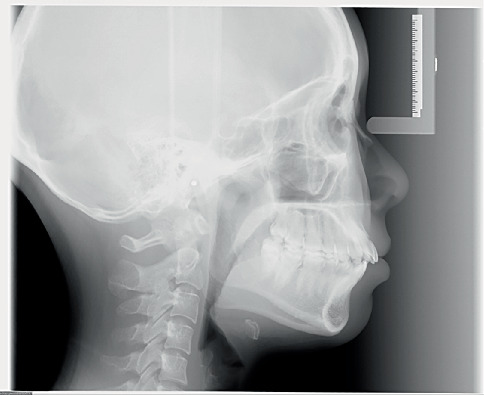
Pretreatment cephalometric x-ray image.

**Figure 4:**
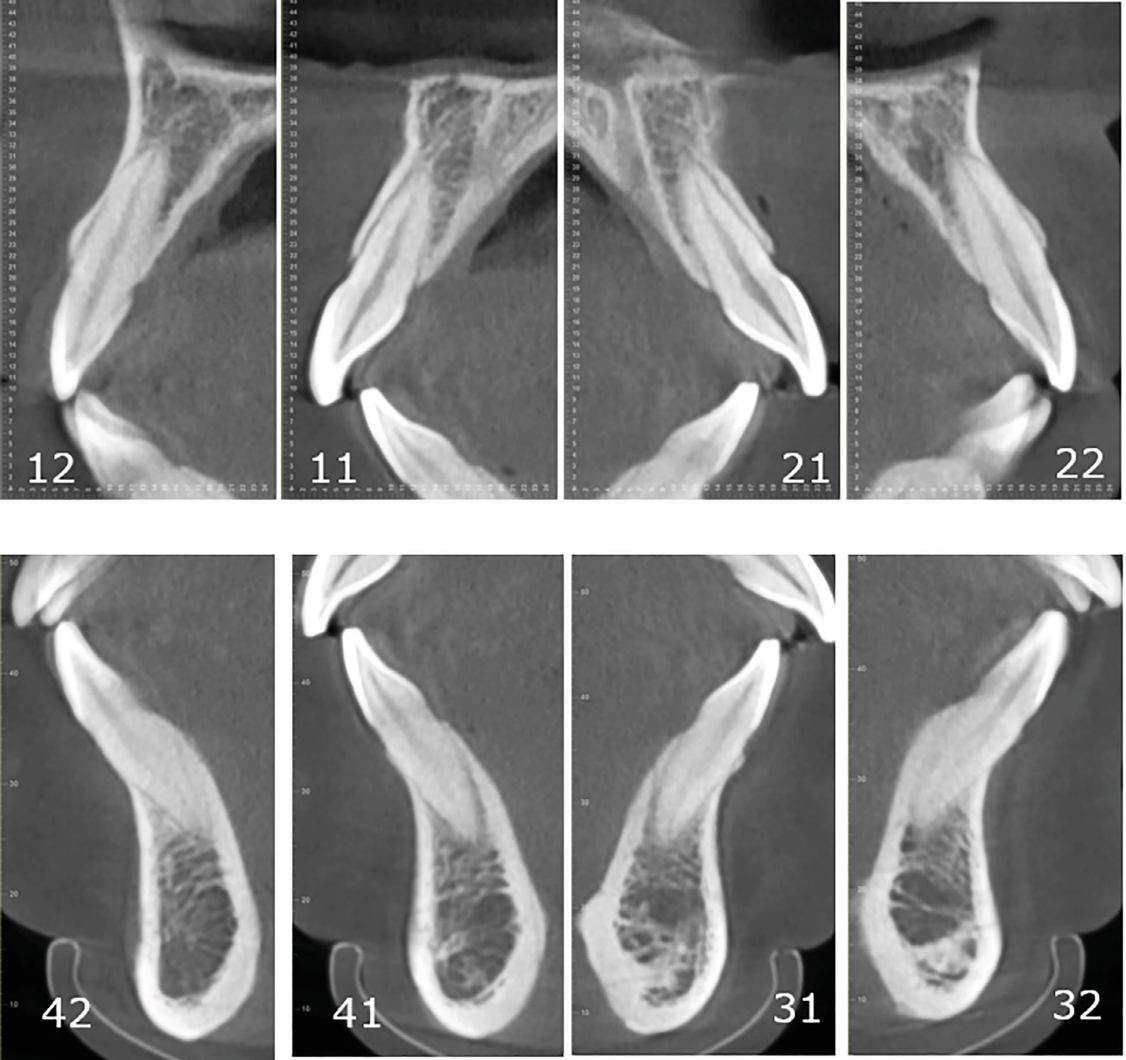
Pretreatment alveolar bone in labiolingual dimension in CBCT.

**Figure 5:**
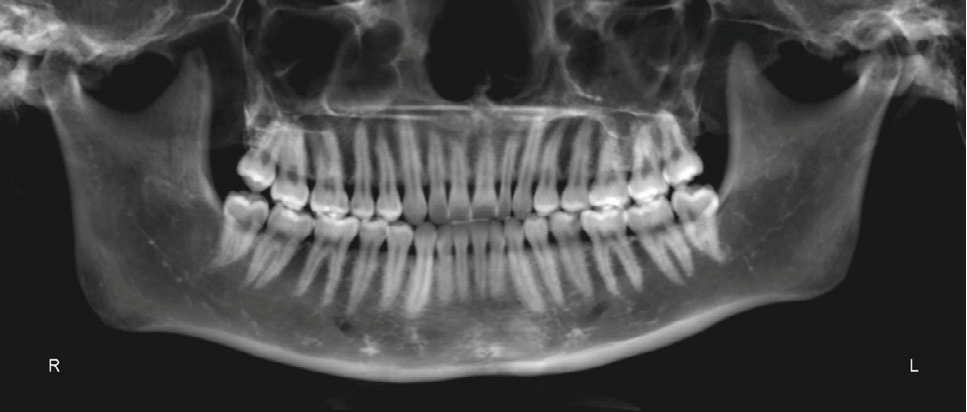
Pretreatment panoramic radiograph.

Pretreatment cephalometric x-ray image and measurements (Figure 3 and Table 1) revealed a mild skeletal Class II malocclusion (A point–nasion–B point [ANB], 5.2°; Wits, 1.6 mm) with a retrognathic mandible (sella–nasion–B point [SNB], 74.5°; gonion–menton/sella–nasion [GoMe/SN], 99.1%), procumbent lower incisors (lower incisor to gonion menton [IMPA], 108.6°; L1-NB, 8 mm), and a protrusive lower lip (lower lip–E line [LL-E], 4.7 mm). CBCT revealed mild alveolar bone resorption and thin alveolar bone in labiolingual dimension of maxillary and mandibular incisors (Figure 4). Panoramic radiography confirmed a full dentition and nonimpacted third molars (Figure 5).

### 2.2. Aims and Objectives

In consideration of mild skeletal deformity of the patient, a camouflage treatment plan was established instead of orthognathic surgery. The treatment goals were to alleviate the tension of lip muscles and improve facial profile. In order to achieve the treatment objectives, four first premolar extractions with reciprocal anchorage were planned to retract the upper and lower incisors.

### 2.3. Treatment Alternatives

In this case, the primary objectives of orthodontic treatment were to retract anterior teeth in both jaws and camouflage protrusion of facial profile. Both fixed appliances and CAs could achieve these goals. Although fixed appliance treatment demonstrates superior performance in torque control, teeth bodily movement, and posterior occlusion [16], adult female patients consistently prioritize esthetic demands during treatment, and CA therapy aligns with these preferences by offering invisible aligners and minimal life quality disruption [17].

Pretreatment evaluation revealed critical periodontal risk factors: thin alveolar bone in anterior regions, close proximity of teeth roots to the cortical plate, thin gingival biotype, and mild alveolar bone resorption with gingival recession. These conditions increased susceptibility to further bone resorption, bone dehiscence, and gingival recession during anterior retraction [18]. CA therapy, predominantly utilizing tipping movements, generates lower and intermittent forces on dentoalveolar structures, reducing cumulative stress on periodontal tissues and enabling physiological remodeling [19]. In addition, fixed appliances exert forces labial to the center of resistance during incisor intrusion, potentially exacerbating labial alveolar bone resorption. This mechanism may induce or worsen anterior “black triangles”, compromising posttreatment miniesthetics [19].

Considering both esthetic priorities and periodontal preservation, CA therapy was selected as the optimal treatment approach for this extraction case.

### 2.4. Treatment Progress

**Figure 6:**
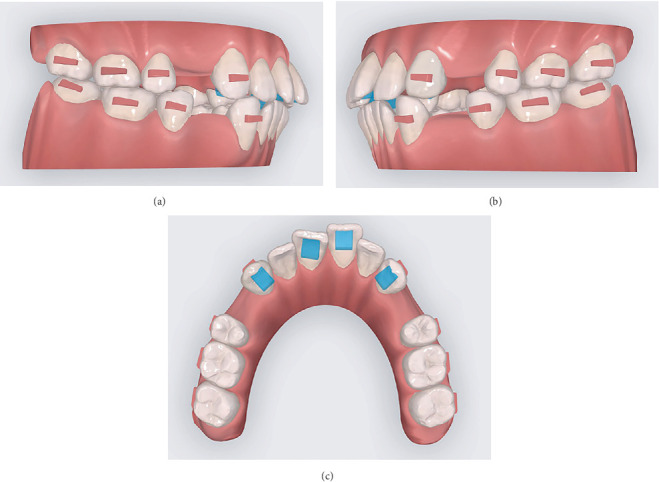
Attachments: (a) right lateral view, (b) left lateral view, and (c) maxillary occlusal view showing guiding planes on Teeth 13, 11, 21, and 23.

**Figure 7:**
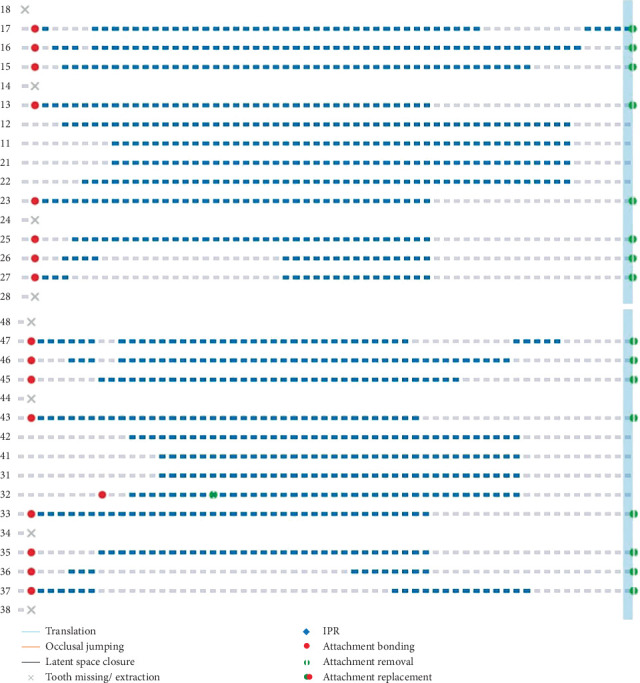
Schematic diagram of maxillary and mandibular tooth movement patterns.

One week after extraction of four first premolors, intraoral scanner (3Shape TRIOS 4; 3Shape A/S, Copenhagen, Denmark) was used to capture digital dental casts. A vacuum-formed retainer was placed immediately following the intraoral scan to prevent displacement of teeth adjacent to the extraction sites during the transition period of aligner design and production. Based on these digital dental casts, CA (Angel Aligner; Angelalgin Technology Inc., Wuxi, China) technicians developed a preliminary tooth movement protocol. Orthodontist then refined this protocol through an online clinician–technician interactive platform system, modifying tooth movement patterns, attachments, and predetermined overcorrection values based on residual space, axial inclination, overbite, overjet, and other indicators to enhance the predictability of tooth movement during treatment. In this case, the protocols were modified twice before aligners were fabricated. The first modification involved changing the double oval attachments on Teeth 13, 23, 33, and 43 to maximum-sized rectangular attachments; converting the vertical rectangular attachments on Teeth 16 and 26 to horizontal rectangular attachments; and adding guiding planes on the maxillary incisors and canines to maintain lower anterior teeth contact with the guiding planes during tooth movement (Figure 6 and Table 2). The second modification involved designing overcorrection values based on teeth displacement distance and steps (Table 3). The definitive protocol was divided into three phases: anchorage preparation of posterior teeth and mesial inclination of canines, distalization of canines and alignment of the anterior teeth, and retraction of anterior teeth with overcorrection designs of anterior teeth intrusion and torque movement (Figure 7).

Following aligner fabrication, attachments were bonded on the corresponding dental position and inspected at Step 0. Guiding planes with extended dimensions are preincorporated into the aligners on maxillary central incisors and canines to ensure mandibular anterior teeth rest onto the surface during movement, facilitating bite opening. The patient was instructed to wear the aligners full-time except during meals and oral hygiene procedures, with a daily wear time exceeding 22 h. Aligners were replaced every 10 days, and follow-up examinations are scheduled approximately every 10 steps.

**Figure 8:**
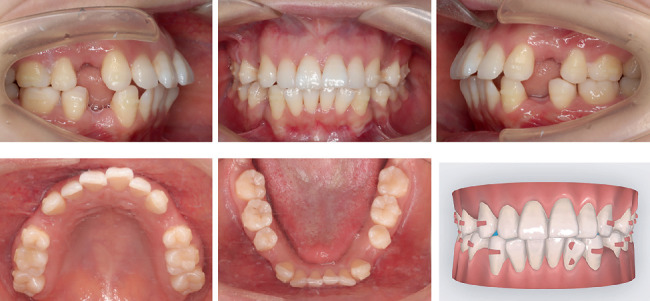
Intraoral photographs and double oval attachment on Tooth 32 at Step 10.

During Steps 0–10, posterior anchorage preparation and mesial tipping of canines were designed. As the mesial tipping of the canines nears completion, simultaneous distal displacement of canines was initiated to create minimal space for alleviating severe crowding of Tooth 32. At the Step 10 follow-up examination, when the aligners exhibited proper fit and sufficient space existed between Teeth 32 and 33, the double oval attachment for Tooth 32 was bonded to assist alignment. The patient was instructed to continue wearing the aligners as prescribed (Figure 8).

During Steps 10–19, distal movement of the canines was continued, and the space created by this movement was utilized to align the upper and lower anterior teeth. In this phase, the extent of canine distal movement exceeded the space required for anterior teeth alignment, resulting in minor residual spacing (approximately 1 mm mesial to the canines) to enhance aligner engagement in the anterior region. At the Step 19 follow-up examination, anterior teeth were fully aligned with appropriately distributed spacing, particularly mesial to the canines. The aligners demonstrated optimal fit, and the attachment on Tooth 32 was removed. The patient was instructed to continue wearing the aligners as before.

**Figure 9:**
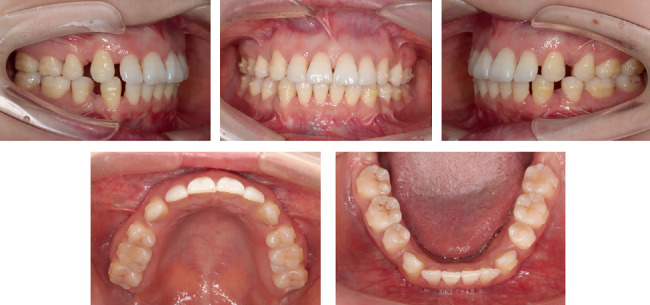
Intraoral photographs at Step 40.

During Steps 19–40, posterior teeth served as anchorage for en masse retraction of anterior teeth. Throughout the retraction phase, intrusion and lingual root torque movement of upper and lower incisors were simultaneously implemented to counteract uncontrolled tipping. Additionally, power ridges were incorporated into the aligners on upper and lower incisors to enhance torque of them and prevent excessive lingual inclination, extrusion of incisors, and associated complications such as deepening overbite and posterior open bite. At the Step 40 examination (Figure 9), the aligners remained fit. Significant reduction in extraction space was observed due to substantial anterior teeth retraction. Posterior anchorage was maintained with no evident mesial tipping. The anterior overbite increased slightly but remained within physiological norm. No significant changes in gingival height or thickness were noted. The patient was instructed to continue wearing the remaining aligners.

During Steps 40–60, the upper and lower incisors were further retracted following the previously established tooth movement pattern to close residual extraction spaces, followed by occlusal adjustment to optimize intercuspation. By completion at Step 60, the treatment goals were achieved after the initial series of aligners, and active orthodontic treatment was concluded with the removal of all composite attachments. In extraction cases, closed extraction sites carry a potential risk of slight space reopening. To maintain treatment outcomes, vacuum-formed retainers were provided with instructions for full-time wear (except during eating and oral hygiene) in the first year, transitioning to nighttime-only wear thereafter.

## 3. Result

**Figure 10:**
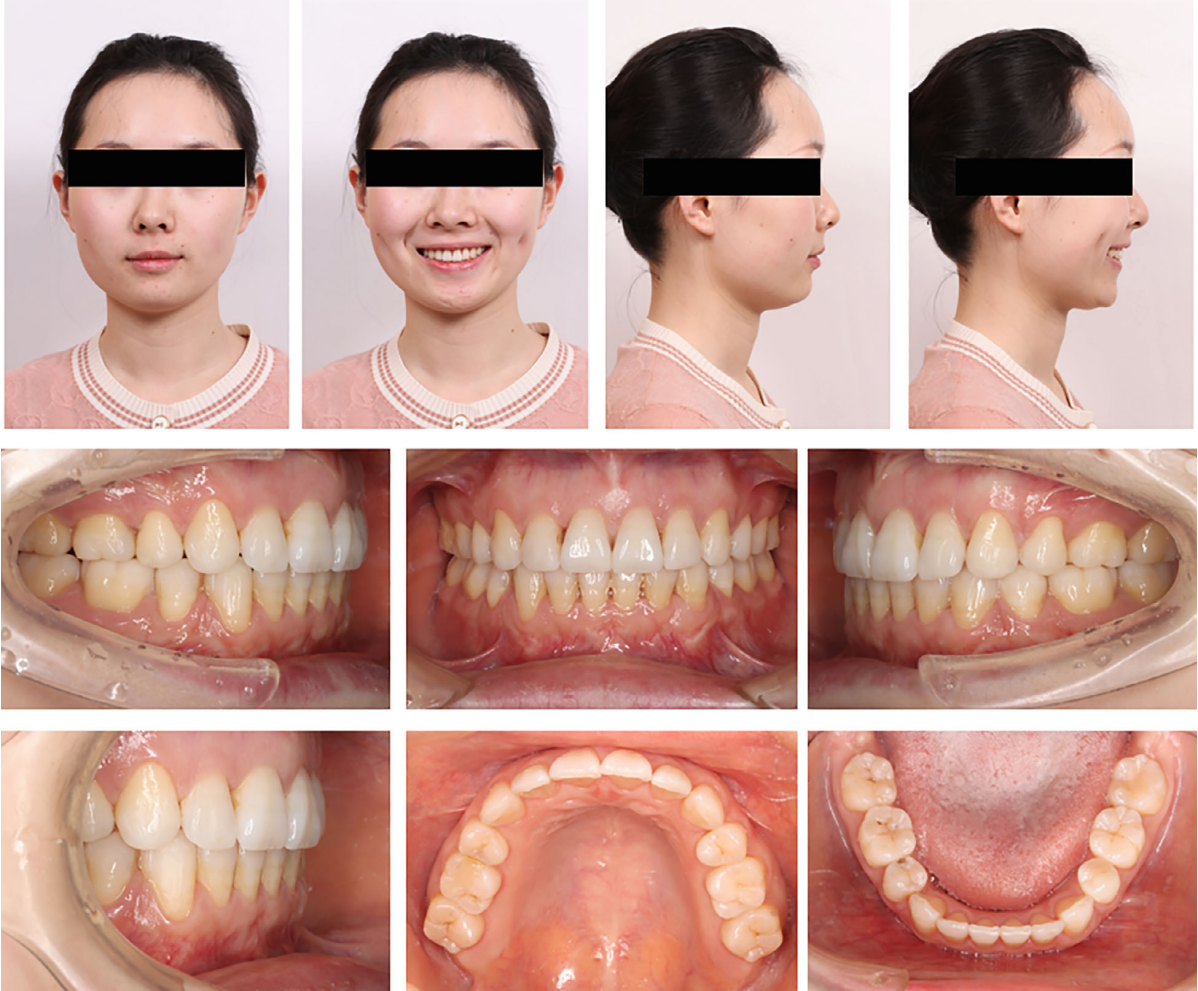
Posttreatment facial and intraoral photographs.

**Figure 11:**
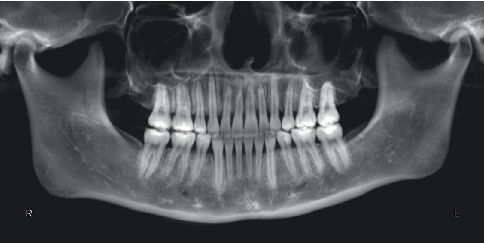
Posttreatment panoramic radiograph.

**Figure 12:**
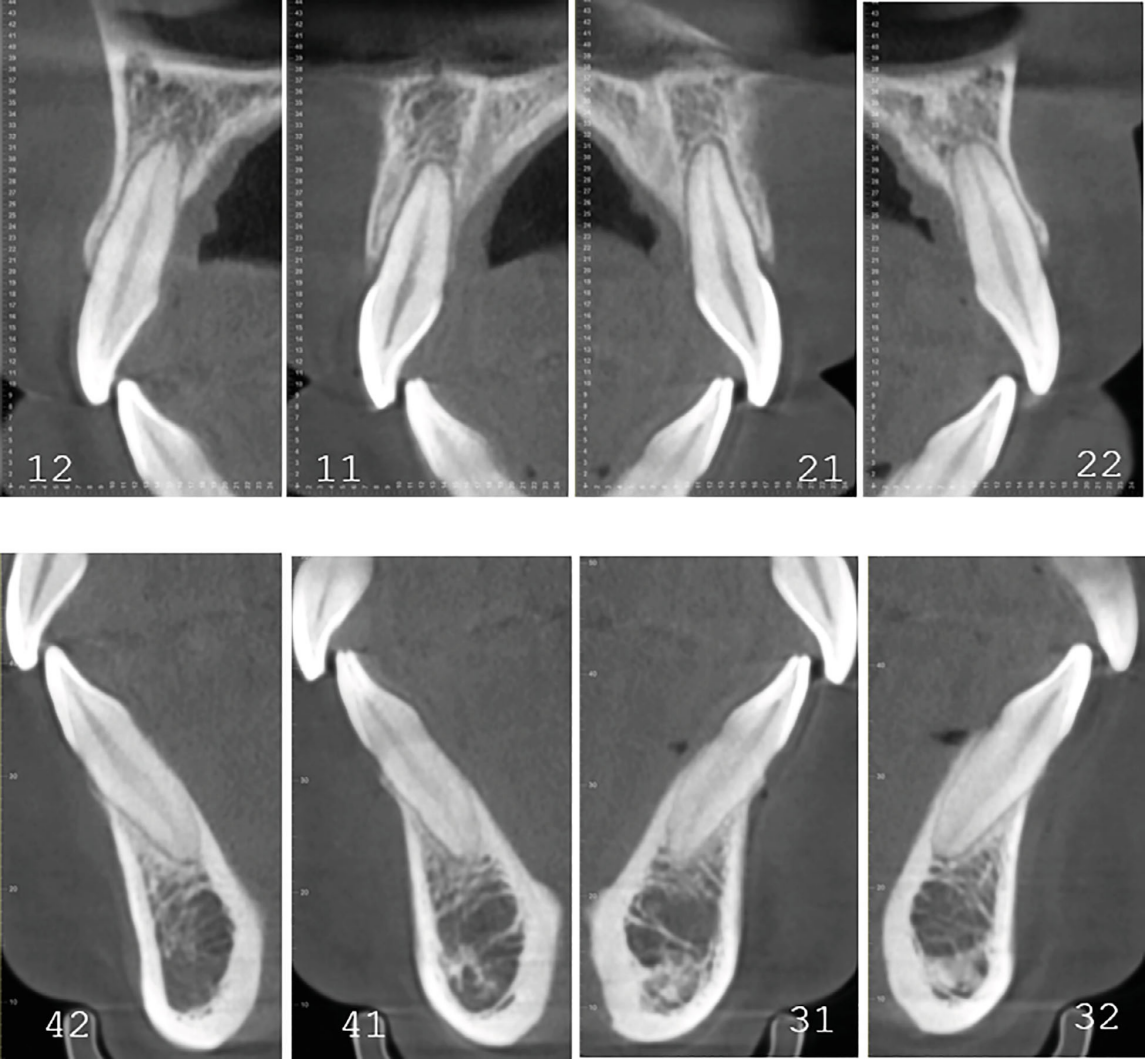
Posttreatment alveolar bone in labiolingual dimension in CBCT.

**Figure 13:**
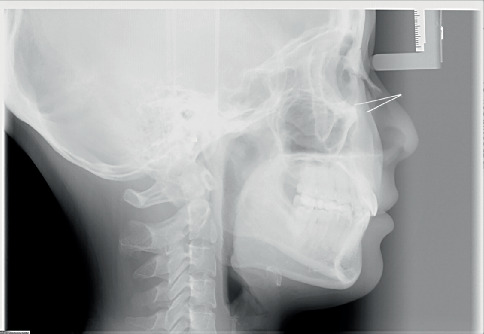
Posttreatment cephalometric x-ray image.

**Figure 14:**
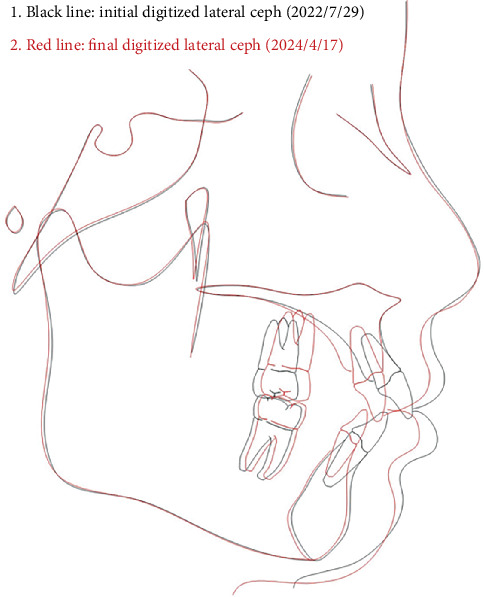
Superimpositions of pre- and posttreatment cephalometric x-ray images (black line: pretreatment; red line: posttreatment).

The patient successfully completed the entire treatment with the initial series of 60 aligners after a total treatment duration of 20 months without experiencing “roller-coaster effects” or midcourse corrections by “off-track”–related refinements. Posttreatment evaluation revealed significant improvements in facial profile and relief of perioral and mentalis muscle tension (Figure 10). Both dentitions achieved full alignment, with bilateral Class I canine and molar relationships, normal anterior overbite and overjet, and tightly interdigitated occlusion. Periodontal assessment showed preserved gingival tissue with no further clinically detectable recession compared to pretreatment records (Figure 10). Posttreatment panoramic radiography demonstrated acceptable root parallelism (Figure 11), while CBCT confirmed preservation of alveolar bone height and thickness without pathologic resorption (Figure 12). Cephalometric measurements (Table 1) of pre- and posttreatment cephalometric x-ray images (Figure 13) and their superimpositions (Figure 14) demonstrated minimal mesial drift of the maxillary and mandibular molars, controlled tipping movement of the incisors: 5.9 mm retraction of upper incisors (U1-NA) accompanied by a 10° reduction in labial inclination, and 4.3 mm retraction of lower incisors (L1-NB) with a 13.3° decrease in proclination. The most significant soft tissue change was a 3.9 mm reduction in the distance from the LL-E, which fell within the normal range posttreatment. These outcomes confirmed precise biomechanical control of tooth movement without adverse root resorption or alveolar bone loss, achieving the predetermined treatment objectives of normalized lip posture, improved facial esthetics, and stable occlusion.

## 4. Discussion

The correction of facial protrusion via premolar extraction and subsequent anterior teeth retraction remains a significant challenge in CA. Both “roller-coaster effects” and “off-track” phenomenon are primary contributors to refinements and even catastrophic failures in CA extraction cases. Frequent refinements not only prolong treatment duration but also increase follow-up visit frequency, clinical chairside time, and healthcare resource utilization. Thus, in complex CA extraction cases, preventing “roller-coaster effects” and “off-track” to minimize refinements—while achieving optimal outcomes with the initial aligner series—represents a critical clinical challenge. Based on studies of recent CA therapy in extraction cases, this case successfully avoided “roller-coaster effects”, “off-track” phenomenon, and subsequent refinements through comprehensive pretreatment analysis, attachment strategies, anchorage control, overcorrection designs, and movement protocols.

To enhance predictability in CA extraction cases, comprehensive diagnostic evaluation and rigorous selection of patients suitable for CA are essential. A study assessing the complexity of CA treatment has shown that difficulty correlates not only with the types of tooth movement required but also with the severity and classification of the patient's pretreatment dentofacial malocclusion and soft tissue esthetics [14]. Pretreatment evaluation demonstrated adequate coordination between the maxillary and mandibular arches in the transverse plane, with the functional cusp width of the maxillary first molars measuring 42.2 mm and the central fossa width of the mandibular first molars at 42.5 mm (Figure 2). In the sagittal plane, cephalometric measurements revealed a mild to moderate Class II skeletal pattern (ANB, 5.2°) and profile protrusion in soft tissue (LL-E, 4.7 mm) with proclined incisors. In the vertical dimension, the pretreatment shallow overbite provided a protective advantage against overbite exacerbation induced by “posterior bite-block effect” [20] and “pendulum effect” [21]. Thus, the mild three-dimensional skeletal discrepancies, coupled with the limited pretreatment soft tissue profile protrusion and favorable dental morphology/proportions for aligner engagement, partially offset the biomechanical challenges. Additionally, the aligner-induced preferential tipping movement and intermittent force delivery promote tissue self-repair mechanisms, effectively reducing periodontal damage and providing critical advantages for this specific case with documented thin labial/buccal bone cortex. These anatomical characteristics served as foundational prerequisites that enabled the successful completion of this CA extraction treatment without refinement phases, achieving optimal outcomes with the initial aligner series.

Attachments enhance the retention of aligners and their control over tooth movement. With advancements in CA technology, the variety of attachments has expanded to include conventional attachments, optimized attachments, and specialized attachments preincorporated into aligners—such as guiding planes and power ridges—to address specific biomechanical demands. In this case, the posterior teeth served as anchorage units to counterbalance the reciprocal forces generated during anterior teeth retraction, necessitating robust aligner retention in the posterior region. Conventional rectangular attachments with beveled edges provide superior retention compared to rounded-edge designs [22]. However, vertical rectangular attachments on posterior teeth compromise gingival seating, reducing movement efficiency and increasing “off-track” risks [9]. Therefore, horizontal rectangular attachments were designed with maximum dimensions tailored to the crown height and morphology of posterior teeth, optimizing the retention stability of aligners. In the anterior region, the canines serve as the primary anchorage for controlling incisor torque and intrusion. Therefore, reciprocal forces generated during incisor movement may compromise aligner–tooth fit at the canines, leading to inadequate force delivery. To mitigate this, a combined design of rectangular attachments on canines and power ridges on incisors was implemented, enhancing anterior intrusion and root control while maintaining the retention of aligners to teeth at canines [6, 23, 24]. The maxillary aligners incorporated guiding planes on the incisors and canines to counteract the posterior intrusion induced by the aligner's inherent “posterior bite-block effects,” thereby effectively mitigating overbite increase and posterior open bite during anterior retraction. By designing guiding planes on both canines and central incisors with extensive size, this innovative approach ensured continuous vertical control throughout the whole treatment, contributing to the case's excellent vertical dimension maintenance and the favorable posttreatment overbite.

When retracting mandibular incisors using reciprocal anchorage following extraction of the mandibular first premolars, the mesial movement of posterior anchorage teeth accounts for approximately 25% of the total extraction space, while the remaining space is occupied by the distal movement of canines and incisor retraction [25]. In the maxilla, the mesial movement of posterior anchorage teeth is slightly greater but does not exceed one-third of the total extraction space [5]. This case employed reciprocal anchorage instead of miniscrew-supported anchorage primarily based on two clinical considerations. First, the pretreatment evaluation revealed moderate soft tissue profile protrusion with mild dental crowding, where the extraction spaces were predominantly closed through anterior retraction [9]. According to the space distribution ratio characteristics of reciprocal anchorage systems, this approach could effectively achieve the therapeutic objectives of anterior retraction and camouflage of profile convexity. Second, the patient's alveolar bone thickness limitations precluded more extensive retraction of incisors. Therefore, anchorage selection should involve a comprehensive evaluation of case-specific characteristics, dental crowding severity, extraction space dimensions, and the dynamic magnitude of teeth displacement during treatment. Strategic implementation and timely modification of anchorage are essential based on biomechanical feedback to achieve desired tooth movement objectives.

**Figure 15:**
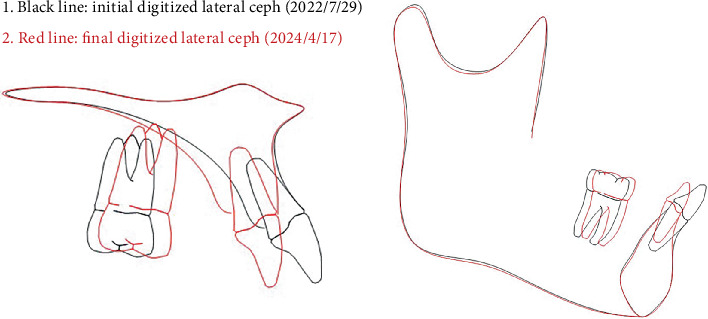
Superimpositions of the maxilla and mandible before and after treatment (black line: pretreatment; red line: posttreatment).

To mitigate the “roller-coaster effects” during the retraction of incisors in extraction cases, overcorrection designs, including posterior anchorage preparation, canine mesial inclination, and anterior torque and intrusion, are imperative. These compensatory biomechanical designs partially counteract the adverse tooth tipping and “roller-coaster effects” during anterior teeth retraction. This case implemented the identical biomechanical strategies to prevent mesial tipping of posterior teeth, distal inclination of canines, and subsequent suboptimal root parallelism. However, in a specific extraction case with CA, determining the optimal magnitude of overcorrection remains a persistent clinical dilemma. Although existing studies have proposed various recommended values for posterior anchorage preparation, these predetermined values demonstrate significant variability across different studies [7, 9, 12], limiting the clinical applicability for precise anchorage preparation quantification in individualized treatment planning. In clinical reality, the design of anchorage preparation magnitude for specific cases should correlate with arch length reduction after preliminary teeth alignment and the number of aligner steps [26]. Qiang et al. [26] demonstrated that for every 0.25 mm of maxillary arch shortening, the canines exhibit 0.2966° of distal inclination, while the second premolars, first molars, and second molars show mesial inclinations of 0.2859°, 0.1888°, and 0.1466°, respectively. Based on these findings, in this case with residual extraction spaces of 6.6 mm (left) and 6.7 mm (right) after preliminary teeth alignment, the predetermined overcorrection values required to counteract dental inclination were calculated as 7.8° mesial inclination for canines and 7.5°, 5.0°, and 3.9° distal inclinations for second premolars, first molars, and second molars, respectively. Based on this foundation, the predetermined overcorrection values should be further supplemented and adjusted according to the initial mesiodistal axial inclination of teeth, crown morphology, and crown–root ratio characteristics to establish the final overcorrection parameters (Table 3). The same theoretical principles and methodological approaches were applied to preset overcorrection values for mandibular posterior anchorage preparation and canine mesial inclination. Superimposition showed the overcorrection design effectively controlled occlusal planes, mimicking fixed appliance outcomes [27], though not in the maxillary anterior region (Figure 15 and Table 4).

**Figure 16:**
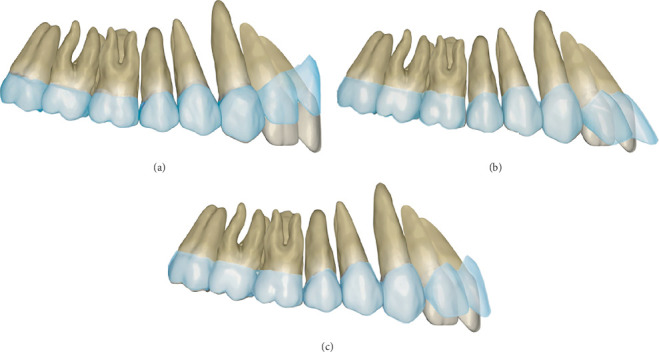
Schematic diagram of the cumulative differences accumulated between the aligners and teeth under different overcorrection preset methods: (a) pure intrusion overcorrection design, (b) pure torque overcorrection design, and (c) combined intrusion–torque overcorrection design.

Uncontrolled tipping movement of incisors may lead to excessive lingual inclination and extrusion of anterior teeth, accompanied by posterior intrusion and deepening of overbite, compromising posttreatment esthetic outcomes and stability. Therefore, predetermined overcorrection of incisor intrusion and lingual root torque was incorporated during anterior retraction. Predetermined overcorrection of anterior intrusion and lingual root torque both can effectively control incisor torque and overbite [28, 29]. Given the relatively low efficiency in achieving pure intrusion or torque movements, isolated design of either intrusion or torque overcorrection for controlled root movement risks significant cumulative discrepancies between the aligners and teeth morphology in a single plane, increasing the likelihood of disengagement (Figure 16). This case combined both intrusion and lingual torque overcorrection, with predetermined magnitudes of intrusive displacement and torque compensation maintained at approximately equivalent levels, thereby minimizing the risk of dimensional mismatch accumulation and subsequent “off-track.” It should be noted that initial incisor labial inclination significantly influences sagittal and vertical displacement during retraction. Greater labial inclination of incisors amplifies the “pendulum effect” during retraction, characterized by reduced retraction magnitude, increased vertical extrusion, and enhanced intrusion tendency from canines to molars [10]. In clinical practice, preset intrusion and lingual root torque compensation values for incisors should be modified according to the initial labial inclination of incisors to achieve the desired final labiolingual inclination.

En masse retraction was employed during the anterior teeth retraction because of superior torque control with less lingual inclination compared to stepwise retraction [4]. Additionally, incorporating 0.75 mm interproximal space between anterior teeth during en masse retraction enhanced aligner engagement and control in anterior regions, thereby promoting bodily tooth movement [30]. In this case, prior to en masse retraction of anterior teeth, the canines were distally moved by 1.1 and 1.5 mm, respectively. The slightly larger distalization spaces compared to the recommendation values served dual purposes: first, addressing mild crowding in both arches, and second, compensating for the limited efficiency of canine distalization in CA extraction therapy. Throughout active retraction, 0.8–1.5 mm interproximal spaces were maintained between canines and lateral incisors in both arches, ensuring optimal aligner–tooth contact for effective force delivery. Therefore, the optimal design approach for anterior retraction in extraction cases integrates predetermined overcorrection of incisor intrusion and lingual root torque, en masse retraction of anterior teeth, and maintenance of interincisal space.

Although integrating existing evidence has helped avoid common adverse outcomes and achieve excellent results in this extraction case treated with CAs, this modality still faces several inherent challenges. These include the material limitations of the aligners themselves, obscure biomechanical mechanisms, inefficient translational tooth movement, and unpredictable treatment outcomes [31, 32]. Enhancing the mechanical properties of the materials, delineating the biomechanical mechanisms of CAs, and further improving the predictability of CA therapy in extraction cases remain issues that need to be addressed.

## 5. Conclusion

According to current research, in extraction therapy with CA, rational case analysis and selection, attachment designs, anchorage control, overcorrection strategies, and tooth movement protocols can effectively minimize the “roller-coaster effects,” enhance the predictability of tooth displacement, and prevent aligner disengagement followed by subsequent treatment refinements. Although individual patient conditions vary and not all cases can achieve single-phase treatment completion, orthodontists must stay updated with emerging research findings to deliver evidence-based treatments and improve clinical outcomes for patients.

## Figures and Tables

**Table 1:** Cephalometric measurements.

**Measurements**	**Pretreatment**	**Posttreatment**	**Norm**
Skeletal analysis			
SNA (°)	79.7	80.1	82.0 ± 3.5
SNB (°)	74.5	74.7	80.0 ± 3.0
ANB (°)	5.2	5.1	2.7 ± 2.0
GoMe/SN (%)	99.1	99.6	112 ± 7.53
Wits (mm)	1.6	1.6	−1.5 ± 2.1
FMA (°)	23.1	22.4	26.0 ± 4.5
SGo/NMe (%)	67.8	67.4	59–63
ODI	84.8	84.1	72.83 ± 5.2
Dental analysis			
U1-SN (°)	103.8	94.1	102.1 ± 5.5
U1-NA (°)	24.1	14.1	22.8 ± 5.7
U1-NA (mm)	5.3	−0.6	4.3 ± 2.7
L1-NB (°)	36.5	23.2	30.3 ± 5.8
L1-NB (mm)	8.0	3.7	4.0 ± 1.8
IMPA (°)	108.6	95.3	93.9 ± 6.2
U1-L1 (°)	114.2	137.3	130.0 ± 6.0
Soft tissue analysis			
Nasolabial angle (°)	118.0	115.9	90–120
UL-E (mm)	0.9	−2.4	−1.5 ± 2.0
LL-E (mm)	4.7	0.8	−2.0 ± 2.0

Abbreviations: ANB, A point–nasion–B point; FMA, gonion–menton to Frankfort horizontal plane; GoMe, gonion–menton; IMPA, lower incisor to gonion–menton; L1-NB, lower incisor to nasion–B point; LL-E, lower lip to E line; NMe, nasion–menton; ODI, overbite depth indicator; SGo, sella–gonion; SN, sella–nasion; SNA, sella–nasion–A point; SNB, sella–nasion–B point; U1-L1, upper incisor to lower incisor; U1-NA, upper incisor to nasion–A point; U1-SN, upper incisor to sella–nasion; UL-E, upper lip to E line.

**Table 2:** Attachment modification.

	**Initial attachment**	**Modified attachment**	**Biomechanical purposes**
13	Wedge attachment	Maximum-sized rectangular attachment	Resisting incisor intrusion-induced aligner lift-off at canines
23	Wedge attachment	Maximum-sized rectangular attachment	
33	Double oval attachment	Maximum-sized rectangular attachment	
43	Double oval attachment	Maximum-sized rectangular attachment	

16	Dual-vertical rectangular attachment	Horizontal rectangular attachment	Superior aligners' gingival margin seating preservation
26	Dual-vertical rectangular attachment	Horizontal rectangular attachment	

Guiding planes	None	Guiding planes on incisors and canines	Prevention of posterior intrusion

**Table 3:** Prescribed orthodontic teeth movement record.

		**17**	**16**	**15**	**13**	**12**	**11**	**21**	**22**	**23**	**25**	**26**	**27**
Translation	E/I (mm)	0.4I	0.2E	0.2E	1.6I	2.8I	3.9I	5.5I	3.2I	2.1 I	0.0	0.3E	0.5I
Translation	La/B/Li (mm)	0.9B	0.9Li	2.0Li	3.3Li	5.0Li	6.8Li	6.7Li	6.3Li	2.6Li	1.2Li	0.1Li	0.4B
Translation	M/D (mm)	0.3M	0.2M	0.0M	5.3D	3.1D	1.0M	0.4D	3.7D	6.4D	0.2M	0.2M	0.1M
Rotation	M/D	5.8° M	8.4° M	5.5° M	4.4° D	1.7° M	15.3° D	7.8° D	0.7° M	12.6° M	13.0° M	11.1° M	7.5° M
Inclination	M/D	3.9° D	5.9° D	8.0° D	8.7° M	1.4° D	4.0° D	1.9° D	4.0° D	7.9° D	7.5° D	6.3° D	4.3° D
Torque	La/B/Li	1.0° B	2.4° B	2.4° Li	1.4° La	4.3° La	5.4° La	3.4° Li	6.3° La	8.7° La	0.5° Li	1.3° B	0.7° Li

		**47**	**46**	**45**	**43**	**42**	**41**	**31**	**32**	**33**	**35**	**36**	**37**

Translation	E/I (mm)	0.2I	0.2E	0.0	2.4I	3.5I	3.4I	3.3I	3.0I	2.5I	0.4E	0.1E	0.7I
Translation	La/B/Li (mm)	0.7Li	1.6Li	2.5Li	4.9Li	4.7Li	4.5Li	4.2Li	5.5Li	5.2Li	0.9Li	0.3Li	0.5Li
Translation	M/D (mm)	1.5M	1.1M	1.8M	3.4D	0.5M	0.7M	1.2D	1.3D	4.3D	0.7M	0.1M	0.3M
Rotation	M/D	5.6° M	7.6° M	3.6° M	15.8° D	27.2° D	4.0° M	9.1° D	35.9° D	30.7° D	14.9° D	3.0° D	3.3° D
Inclination	M/D	14.1° D	9.0° D	12.0° D	12.3° M	7.5° M	1.2° D	0.9° M	10.1° M	12.2° M	8.5° D	6.8° D	10.9° D
Torque	La/B/Li	9.2° B	1.6° Li	1.2° Li	6.7° Li	8.8° Li	6.1° Li	4.9° Li	10.3° Li	10.7° Li	12.0° Li	2.3° Li	2.2° B

Abbreviations: B, buccal; D, distal; E, extrusion; I, intrusion; La, labial; Li, lingual; M, mesial.

**Table 4:** Occlusal plane changes pre- versus posttreatment.

		**Pretreatment**	**Posttreatment**	**Difference**	**Reference**
Maxillary	BAOP	186.5	171.5	−15.0	−9.91 ± 6.08
	AOP-FH	170.9	160.3	10.6	−9.18 ± 4.43
	POP-FH	15.6	14.8	−0.8	−0.73 ± 3.34

Mandibular	BAOP	172.5	172.4	0.1	−7.48 ± 8.28
	AOP-MP	165.7	161.5	−4.2	−8.47 ± 7.83
	POP-MP	7.8	10.9	2.1	0.99 ± 3.44

Abbreviations: AOP, anterior occlusal plane; BAOP, bending angle of occlusal plane; FH, Frankfort horizontal plane; MP, mandibular plane; POP, posterior occlusal plane.

## Data Availability

The data that support the findings of this study are available from the corresponding author upon reasonable request.
